# Systemic Lactate Dehydrogenase Levels as a Predictor of Progression from Non-Proliferative to Proliferative Diabetic Retinopathy

**DOI:** 10.3390/jcm14248696

**Published:** 2025-12-08

**Authors:** Esraa Shosha, Muhammad Z. Chauhan, Jawad Muayad, Ahmed B. Sallam, Abdelrahman Y. Fouda

**Affiliations:** 1 Department of Pharmacology and Toxicology, College of Medicine, University of Arkansas for Medical Sciences, Little Rock, AR 72205, USA; efshosha@uams.edu; 2 Clinical Pharmacy Department, Cairo University, Cairo 11562, Egypt; 3 Department of Ophthalmology, University of Arkansas for Medical Sciences, Little Rock, AR 72205, USA; mzchauhan@uams.edu; 4 College of Medicine, Texas A&M University, Houston, TX 77843, USA; jawad.muayad@gmail.com; 5 Department of Ophthalmology, Ain Shams University, Cairo 11517, Egypt

**Keywords:** proliferative diabetic retinopathy, non-proliferative diabetic retinopathy, lactate dehydrogenase, biomarker

## Abstract

**Objective:** Diabetic retinopathy (DR) is a leading cause of blindness, and understanding its progression from non-proliferative (NPDR) to sight-threatening proliferative diabetic retinopathy (PDR) is crucial. Systemic lactate dehydrogenase (LDH) has been implicated in various disease processes. We investigated the association between systemic LDH levels at the time of NPDR diagnosis and the 1-year risk of progression to PDR and its complications. **Methods:** We conducted a retrospective, propensity-matched cohort study using the TriNetX US Collaborative Network. Patients with type 2 diabetes and a new diagnosis of NPDR were stratified into three groups based on a single LDH measurement taken within 6 months of the index date: low (<200 U/L), moderate (201–280 U/L), and high (≥281 U/L). Two separate analyses were performed: one comparing the low-LDH group to the moderate-LDH group, and another comparing the low-LDH group to the high-LDH group. The primary outcomes were the 1-year absolute risks and risk ratios (relative risk, RR) for PDR, tractional retinal detachment (TRD), and vitreous hemorrhage (VH). **Results:** Comparing the low-LDH cohort to the moderate-LDH cohort, the moderate-LDH group had a higher 1-year absolute risk of PDR (3.93% vs. 2.96%), TRD (1.35% vs. 0.99%), and VH (4.38% vs. 3.51%). Comparing the low-LDH group to the high-LDH group, the high-LDH cohort showed an increased risk for PDR (3.66% vs. 3.00%), TRD (1.27% vs. 0.96%), and VH (1.27% vs. 0.96%). **Conclusions:** Our findings demonstrate a consistent, dose-dependent relationship between higher systemic LDH levels and an increased risk of progression to PDR and its complications.

## 1. Introduction

Diabetic retinopathy (DR) is the most common microvascular complication of diabetes mellitus and a leading cause of new-onset blindness in working-age adults worldwide. The disease is characterized by a spectrum of vascular changes, beginning with non-proliferative diabetic retinopathy (NPDR), which presents as microaneurysms, intraretinal hemorrhages and exudates, and potentially advancing to the more severe sight-threatening stage of proliferative diabetic retinopathy (PDR), characterized by neovascularization. PDR can lead to subsequent complications such as vitreous hemorrhage (VH) and tractional retinal detachment (TRD) [1]. Studies have estimated the median time to progression from moderate NPDR to PDR to be around 2 years [2], yet this varies widely depending on the stage of NPDR, with up to 50 to 75% of eyes with severe NPDR progressing to PDR within one year [3,4].

The pathogenesis of DR progression is complex and involves multiple factors, including chronic hyperglycemia, inflammation, and cellular hypoxia [5]. Lactate dehydrogenase (LDH) is a ubiquitous intracellular enzyme that plays a crucial role in glycolysis. Elevated systemic LDH levels in the bloodstream are often considered a non-specific marker of tissue damage, cellular turnover, or hypoxia [6]. Previous studies investigated the association between serum LDH levels and DR, indicating a strong association between elevated LDH levels (> 134 U/L) and DR [7,8]. However, its specific role as a predictive biomarker for the progression of NPDR to PDR and in evaluating TRD and VH has not been studied in large patient population studies.

This study aimed to investigate whether varying systemic LDH levels at the time of NPDR diagnosis are associated with a higher 1-year risk of progression to PDR, TRD, or VH in type 2 diabetic patients. We hypothesized that patients with higher LDH levels would be at increased risk for these adverse outcomes, even after accounting for known risk factors and comorbid conditions.

## 2. Materials and Methods

### 2.1. Study Design and Data Source

This was a retrospective, propensity-matched cohort study utilizing data from the TriNetX US Collaborative Network (Cambridge, MA, USA), a large-scale research database of electronic health records from numerous healthcare organizations. The network includes >90 healthcare organizations (HCOs) in the United States and abroad, with >130 million patients. The study period encompassed a 1-year follow-up from the index date for each patient. The University of Arkansas for Medical Sciences Institutional Review Board (UAMS IRB) waived the study review/approval since the patient data were deidentified. The study adhered to the principles outlined in the Declaration of Helsinki.

### 2.2. Patient Cohorts and Exposure Definition

All patients included in this cohort were diagnosed with type 2 diabetes mellitus. The study cohort included patients with a first encounter coded for a diagnosis of NPDR, which served as the index date. For each patient, a single LDH measurement was retrieved if it was recorded within six months before or after the index date. Based on this measurement, patients were assigned to one of three mutually exclusive strata: low LDH: LDH levels < 200 Units per Liter (U/L); moderate LDH: LDH levels = 201–280 U/L, and high LDH: LDH levels ≥ 281 U/L. To ensure a clean separation between groups, any patient who had ever recorded an LDH value outside the qualifying range of their assigned stratum was excluded from the analysis.

### 2.3. Propensity Score Matching

We conducted two separate 1:1 greedy nearest-neighbor propensity score–matched comparisons. The first comparison was between patients with low LDH and those with moderate LDH, in which those with LDH < 200 U/L were matched to those with LDH 201–280 U/L (baseline characteristics are shown in Table 1). The second comparison compared low LDH vs. high LDH, matching patients with LDH < 200 U/L to those with LDH ≥281 U/L (baseline characteristics are shown in Table 2). The matching process was designed to balance baseline characteristics and minimize confounding factors that may affect the study outcomes. Covariates included demographics such as current age, age at NPDR diagnosis, sex, race, and Hispanic ethnicity. Metabolic and vascular comorbidities included HbA1c, body mass index (BMI), hypertension, dyslipidemia, and proteinuria. The ocular disease stage was accounted for using NPDR severity codes, the presence of macular edema, and open-angle glaucoma. Prior ophthalmic procedures were also included, specifically codes for intravitreal injections and other ophthalmic interventions, with the complete list of codes provided in (Table 3).

## 3. Outcomes

The primary outcomes, assessed within one year of the index date, were the absolute risk and relative risk of developing PDR, VH, and TRD. We calculated the risk ratios (also referred to as relative risk or RR) and their corresponding 95% confidence intervals (CIs) for each comparison. We set the significance level for this study at a *p*-value < 0.05 using two-sided tests.

## 4. Results

### 4.1. Comparison of Low (<200 U/L) vs. Moderate (201–280 U/L) LDH Cohorts

**Figure 1:**
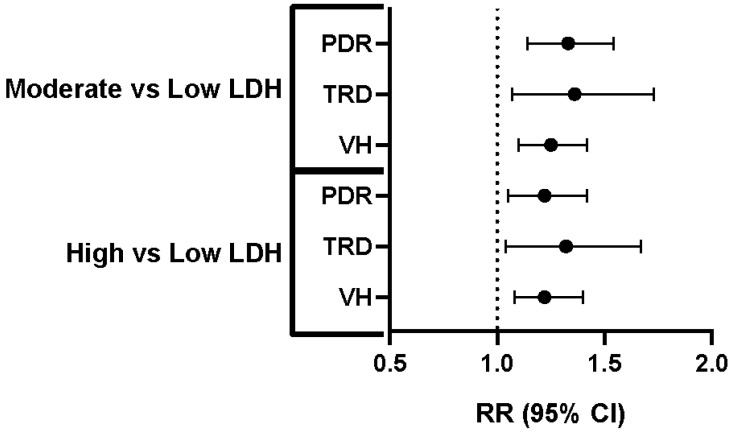
Increased Risk of PDR, TRD, and VH with Higher LDH Levels. Relative risk (RR) and 95% confidence intervals (CI) for proliferative diabetic retinopathy (PDR), tractional retinal detachment (TRD), and vitreous hemorrhage (VH) in individuals with moderate versus low lactate dehydrogenase (LDH) levels and high versus low LDH levels. The dashed vertical line represents an RR of 1.0, indicating no difference in risk compared to the reference group (low LDH). Each horizontal line represents the 95% CI for the RR, with the dot indicating the point estimate of the RR. For all three complications (PDR, TRD, and VH), both the “moderate vs. low LDH” and “high vs. low LDH” groups show RR greater than 1.0, indicating a statistically significant increased risk of PDR, TRD, and VH in individuals with moderate and high LDH levels compared to those with low LDH levels.

After matching, the cohorts were well-balanced on all predefined covariates (Table 1). For PDR, the 1-year absolute risk of PDR was 2.96% in the low-LDH cohort and 3.93% in the moderate-LDH cohort. This represented a 33% increase in relative risk (RR 1.33, 95% CI 1.14–1.54). TRD occurred in 0.99% of the low-LDH cohort and 1.35% of the moderate-LDH cohort. The relative risk was 1.36 (95% CI 1.07–1.73). VH occurred in 3.51% of the low-LDH cohort and 4.38% of the moderate-LDH cohort, resulting in a relative risk of 1.25 (95% CI 1.10–1.42) (Table 4 and Figure 1). In this comparison, a modest elevation of LDH levels from <200 U/L to 201–280 U/L was associated with small but statistically significant absolute risk increments of 0.4 to 0.9 percentage points for all three sight-threatening outcomes.

### 4.2. Comparison of Low (<200 U/L) vs. High (≥281 U/L) LDH Cohorts

After matching, the cohorts were well-balanced on all predefined covariates (Table 2). This analysis also showed well-balanced cohorts after matching propensity scores. In the high-LDH cohort, the 1-year absolute risk of PDR was 3.66%, compared to 3.00% in the matched controls. The relative risk was 1.22 (95% CI 1.05–1.42). TRD developed in 1.27% of the high-LDH patients versus 0.96% of their matched controls. This corresponds to an RR of 1.32 (95% CI 1.04–1.67). VH occurred in 1.27% of patients with LDH ≥ 281 U/L and 0.96% of the control group, yielding an RR of 1.22 (95% CI 1.08–1.40) (Table 4 and Figure 1).

## 5. Discussion

The results of this study demonstrated a positive and significant association between higher systemic LDH levels and an increased 1-year risk of progression from NPDR to the vision-threatening PDR. Across both propensity-matched comparisons, the direction of effect was uniform: as the LDH levels increased, so did the absolute probability of PDR, TRD, and VH. The observed relative risks for these outcomes ranged from approximately 20% to 35%, despite matching for a wide array of demographic, metabolic, and ocular confounding factors.

LDH is a ubiquitous enzyme crucial for cellular energy metabolism. As an indicator of systemic cell damage, circulating LDH levels are measured in terms of enzymatic activity. While the reference range for adults is typically between 125 and 220 IU/L, this can vary among laboratories. Elevated LDH levels are a non-specific marker for a broad spectrum of pathologies, including tissue injury, malignancy, and blood disorders. Therefore, interpreting LDH activity requires correlation with other clinical and laboratory findings to identify the specific site of cellular necrosis or turnover. For example, a recent study demonstrated that serum LDH activities are correlated with various diseases, with a robust correlation observed in hepatic encephalopathy and lung fibrosis [9]. Our finding suggests that systemic LDH levels may be an independent marker of vulnerability to PDR. The underlying mechanism may be linked to LDH’s role in modulating cellular stress. In the context of diabetic retinopathy, elevated LDH could be a systemic reflection of the microvascular damage, chronic inflammation, and increased anaerobic metabolism occurring within the hypoxic retina. As retinal hypoxia worsens, cells shift towards anaerobic glycolysis, producing lactate and releasing LDH into the bloodstream upon cellular damage, which in turn fuels the neovascularization cascade characteristic of PDR [10,11].

The use of a large, real-world database and a propensity-matched design are key strengths of this study. This approach allowed us to control for numerous potential confounders, lending greater confidence to the observed associations. The consistent dose–response relationship across two separate matched analyses further strengthens our conclusion. In addition, our study is the first to show the association between LDH levels and VH and TRD. A previous study demonstrated the association between the elevated LDH levels (>134 U/L) and the increased risk of DR in type 2 diabetic patients [7]. However, it did not evaluate the risk associated with the different categories of LDH levels and the progression of DR. Serum LDH levels have been extensively studied as a biomarker for various human diseases [9]. It has been detected in other retinal diseases, including retinoblastoma [12]. Our study introduces LDH as a biomarker for the risk of progression from NPDR to PDR, which could help identify patients who require more frequent retinal monitoring to prevent vision loss. However, it is essential to acknowledge the study’s limitations; our study is correlational and cannot establish a causal relationship between LDH and progression from NPDR to PDR. Furthermore, we did not stratify patients by NPDR stage, and the follow-up was limited to one year from the index date. More importantly, LDH can be upregulated in various acute and chronic conditions (e.g., occult malignancy, hemolysis, liver or muscle disease), as mentioned earlier; therefore, it is essential to consider the patients’ comorbidities and repeat measurements after acute conditions that may affect LDH levels subside.

## 6. Conclusions

Systemic lactate dehydrogenase levels, even at modest elevations above the normal range, are significantly and independently associated with a higher risk of developing PDR and its complications (TRD and VH) within one year in type 2 diabetic patients. These findings suggest that LDH could serve as a valuable and accessible biomarker for risk stratification in patients with NPDR.

## Figures and Tables

**Table 1:** Baseline Characteristics of Patients with Non-Proliferative Diabetic Retinopathy: Moderate vs. Low LDH Cohorts Before and After Propensity Score Matching by LDH Level.

	Before Matching			After Matching		
Characteristics	Moderate LDH (n = 14,615)	Low LDH (n = 13,514)	Standardized Difference	Moderate LDH (n = 11,708)	Low LDH (n = 11,708)	Standardized Difference
Age, mean ± SD	63.5 ± 13.5	62.9 ± 13.7	0.0473	63.1 ± 13.5	63.2 ± 13.6	0.0034
Gender, No. (%)						
Male	6878 (49.92%)	6493 (52.94%)	0.0603	6121 (52.28%)	6106 (52.15%)	0.0026
Race, No. (%)						
White	7350 (53.35%)	7120 (58.05%)	0.0947	6708 (57.29%)	6712 (57.33%)	0.0007
Black or African American	3688 (26.77%)	2764 (22.53%)	0.0983	2762 (23.59%)	2734 (23.35%)	0.0056
Asian	554 (4.02%)	512 (4.17%)	0.0077	477 (4.07%)	485 (4.14%)	0.0034
Native Hawaiian or Other Pacific Islander	145 (1.05%)	100 (0.82%)	0.0247	99 (0.85%)	97 (0.83%)	0.0019
American Indian or Alaska Native	133 (0.97%)	93 (0.76%)	0.0224	91 (0.78%)	93 (0.79%)	0.0019
Other Race	631 (4.58%)	568 (4.63%)	0.0024	537 (4.59%)	540 (4.61%)	0.0012
Unknown Race	1277 (9.27%)	1109 (9.04%)	0.0079	1034 (8.83%)	1047 (8.94%)	0.0039
Ethnicity, No. (%)						
Hispanic/Latinx	1791 (13.00%)	1549 (12.63%)	0.0111	1482 (12.66%)	1497 (12.79%)	0.0038
Comorbidities, No. (%)						
Essential (primary) hypertension	11,983 (86.97%)	10,369 (84.53%)	0.0698	10,041 (85.76%)	10,051 (85.85%)	0.0024
Hyperlipidemia	11,076 (80.39%)	9563 (77.96%)	0.0598	9269 (79.17%)	9264 (79.13%)	0.0011
T2DM with mild NPDR without macular edema	4092 (29.70%)	3722 (30.34%)	0.0141	3535 (30.19%)	3502 (29.91%)	0.0061
T2DM with mild NPDR with macular edema	787 (5.71%)	656 (5.35%)	0.0159	631 (5.39%)	639 (5.46%)	0.0030
T2DM with moderate NPDR without macular edema	1423 (10.33%)	1141 (9.30%)	0.0345	1092 (9.33%)	1108 (9.46%)	0.0047
T2DM with moderate NPDR with macular edema	764 (5.55%)	607 (4.95%)	0.0268	585 (5.00%)	592 (5.06%)	0.0027
T2DM with severe NPDR without macular edema	668 (4.85%)	503 (4.10%)	0.0362	474 (4.05%)	489 (4.18%)	0.0065
T2DM with severe NPDR with macular edema	464 (3.37%)	353 (2.88%)	0.0282	348 (2.97%)	346 (2.96%)	0.0010
Primary open-angle glaucoma	717 (5.20%)	543 (4.43%)	0.0363	531 (4.54%)	533 (4.55%)	0.0008
Proteinuria	3442 (24.98%)	2599 (21.19%)	0.0901	2613 (22.32%)	2589 (22.11%)	0.0049
Alcohol dependence	385 (2.79%)	342 (2.79%)	0.0004	320 (2.73%)	327 (2.79%)	0.0036
Tobacco use	783 (5.68%)	679 (5.54%)	0.0064	630 (5.38%)	653 (5.58%)	0.0086
Central retinal artery occlusion	57 (0.41%)	45 (0.37%)	0.0075	42 (0.36%)	43 (0.37%)	0.0014
Retinal artery branch occlusion	54 (0.39%)	49 (0.40%)	0.0012	49 (0.42%)	47 (0.40%)	0.0027
Partial retinal artery occlusion	43 (0.31%)	41 (0.33%)	0.0039	38 (0.33%)	37 (0.32%)	0.0015
Central retinal vein occlusion	199 (1.44%)	160 (1.30%)	0.0120	159 (1.36%)	157 (1.34%)	0.0015
Tributary (branch) retinal vein occlusion	193 (1.40%)	150 (1.22%)	0.0156	150 (1.28%)	145 (1.24%)	0.0038
Low income	68 (0.49%)	41 (0.33%)	0.0248	37 (0.32%)	41 (0.35%)	0.0059
Intravitreal injection	1360 (9.87%)	1005 (8.19%)	0.0585	998 (8.52%)	993 (8.48%)	0.0015
Lab Values, mean ± SD						
Hemoglobin	10.5 ± 2.37	10.9 ± 2.42	0.1826	10.5 ± 2.39	10.9 ± 2.41	0.1474
Hemoglobin A1c	7.68 ± 1.97	7.64 ± 1.96	0.0246	7.69 ± 1.96	7.63 ± 1.96	0.0283
Triglyceride	148 ± 147	154 ± 136	0.0408	150 ± 153	154 ± 135	0.0301
Cholesterol	154 ± 53.7	152 ± 50.3	0.0511	154 ± 52.6	152 ± 50.3	0.0370
Body Mass Index	31.2 ± 7.88	30.8 ± 7.56	0.0461	31.3 ± 7.89	30.8 ± 7.54	0.0597

LDH: Lactate dehydrogenase, SD: standard deviation, No.: Number of patients, T2DM: Type 2 diabetes mellitus, NPDR: Non-proliferative diabetic retinopathy.

**Table 2:** Baseline Characteristics of Patients with Non-Proliferative Diabetic Retinopathy: High vs. Low LDH Cohorts Before and After Propensity Score Matching by LDH Level.

	Before Matching			After Matching		
Characteristics	High LDH (n = 21,915)	Low LDH (n = 13,514)	Standardized Difference	High LDH (n = 12,154)	Low LDH (n = 12,154)	Standardized Difference
Age, mean ± SD	62.6 ± 13.2	62.9 ± 13.7	0.0187	62.9 ± 13.3	62.9 ± 13.7	0.0009
Gender, No. (%)						
Male	10,257 (50.05%)	6493 (52.94%)	0.0577	6468 (53.22%)	6417 (52.80%)	0.0084
Race, No. (%)						
White	10,202 (49.78%)	7120 (58.05%)	0.1664	7087 (58.31%)	7039 (57.92%)	0.0080
Black or African American	6169 (30.10%)	2764 (22.53%)	0.1725	2721 (22.39%)	2757 (22.68%)	0.0071
Asian	909 (4.44%)	512 (4.17%)	0.0129	531 (4.37%)	505 (4.16%)	0.0106
Native Hawaiian or Other Pacific Islander	143 (0.70%)	93 (0.76%)	0.0071	96 (0.79%)	93 (0.77%)	0.0028
American Indian or Alaska Native	199 (0.97%)	100 (0.82%)	0.0166	91 (0.75%)	99 (0.82%)	0.0075
Other Race	862 (4.21%)	568 (4.63%)	0.0207	556 (4.58%)	560 (4.61%)	0.0016
Unknown Race	2009 (9.80%)	1109 (9.04%)	0.0261	1072 (8.82%)	1101 (9.06%)	0.0084
Ethnicity, No. (%)						
Hispanic/Latinx	2355 (11.49%)	1549 (12.63%)	0.0349	1504 (12.38%)	1527 (12.56%)	0.0057
Comorbidities, No. (%)						
Essential (primary) hypertension	18,027 (87.97%)	10,369 (84.53%)	0.0998	10,311 (84.84%)	10,300 (84.75%)	0.0025
Hyperlipidemia	16,602 (81.01%)	9563 (77.96%)	0.0756	9536 (78.46%)	9508 (78.23%)	0.0056
T2DM with mild NPDR without macular edema	5704 (27.83%)	3722 (30.34%)	0.0553	3703 (30.47%)	3673 (30.22%)	0.0054
T2DM with mild NPDR with macular edema	980 (4.78%)	656 (5.35%)	0.0258	663 (5.46%)	649 (5.34%)	0.0051
T2DM with moderate NPDR without macular edema	1958 (9.55%)	1141 (9.30%)	0.0086	1166 (9.59%)	1125 (9.26%)	0.0115
T2DM with moderate NPDR with macular edema	928 (4.53%)	607 (4.95%)	0.0198	608 (5.00%)	600 (4.94%)	0.0030
T2DM with severe NPDR without macular edema	951 (4.64%)	503 (4.10%)	0.0264	501 (4.12%)	501 (4.12%)	0.0000
T2DM with severe NPDR with macular edema	589 (2.87%)	353 (2.88%)	0.0002	366 (3.01%)	351 (2.89%)	0.0073
Primary open-angle glaucoma	1044 (5.09%)	543 (4.43%)	0.0314	516 (4.25%)	542 (4.46%)	0.0105
Proteinuria	5033 (24.56%)	2599 (21.19%)	0.0803	2598 (21.38%)	2591 (21.32%)	0.0014
Alcohol dependence	720 (3.51%)	342 (2.79%)	0.0415	323 (2.66%)	340 (2.80%)	0.0086
Tobacco use	1155 (5.64%)	679 (5.54%)	0.0044	678 (5.58%)	678 (5.58%)	0.0000
Central retinal artery occlusion	86 (0.42%)	45 (0.37%)	0.0084	43 (0.35%)	45 (0.37%)	0.0027
Retinal artery branch occlusion	74 (0.36%)	49 (0.40%)	0.0062	46 (0.38%)	49 (0.40%)	0.0040
Partial retinal artery occlusion	65 (0.32%)	41 (0.33%)	0.0030	39 (0.32%)	41 (0.34%)	0.0029
Central retinal vein occlusion	243 (1.19%)	160 (1.30%)	0.0107	155 (1.28%)	157 (1.29%)	0.0015
Tributary (branch) retinal vein occlusion	252 (1.23%)	150 (1.22%)	0.0006	149 (1.23%)	146 (1.20%)	0.0023
Low income	86 (0.42%)	41 (0.33%)	0.0139	38 (0.31%)	41 (0.34%)	0.0043
Intravitreal injection	1779 (8.68%)	1005 (8.19%)	0.0175	991 (8.15%)	1000 (8.23%)	0.0027
Lab Values, mean ± SD						
Hemoglobin	10.1 ± 2.32	10.9 ± 2.42	0.3197	10.2 ± 2.34	10.9 ± 2.42	0.2917
Hemoglobin A1c	7.7 ± 2.01	7.64 ± 1.96	0.0340	7.69 ± 1.99	7.64 ± 1.96	0.0256
Triglyceride	155 ± 129	154 ± 136	0.0081	157 ± 131	154 ± 136	0.0202
Cholesterol	154 ± 54.2	152 ± 50.3	0.0466	152 ± 53.7	152 ± 50.4	0.0048
Body Mass Index	31 ± 7.79	30.8 ± 7.56	0.0169	30.9 ± 7.69	30.8 ± 7.55	0.0091

LDH: Lactate dehydrogenase, SD: standard deviation, No.: Number of patients, T2DM: Type 2 diabetes mellitus, NPDR: Non-proliferative diabetic retinopathy.

**Table 3:** Codes Used in the Study.

Code System	Code	Description
ICD-10-CM	I10	Essential (primary) hypertension
ICD-10-CM	E78	Disorders of lipoprotein metabolism and other lipidemias
ICD-10-CM	E11.329	Type 2 diabetes mellitus with mild NPDR without macular edema
ICD-10-CM	R80	Proteinuria
ICD-10-CM	E11.339	Type 2 diabetes mellitus with moderate NPDR without macular edema
ICD-10-CM	E11.321	Type 2 diabetes mellitus with mild NPDR with macular edema
ICD-10-CM	Z72.0	Tobacco use
ICD-10-CM	E11.331	Type 2 diabetes mellitus with moderate NPDR with macular edema
ICD-10-CM	H40.11	Primary open-angle glaucoma
ICD-10-CM	E11.349	Type 2 diabetes mellitus with severe NPDR without macular edema
ICD-10-CM	E11.341	Type 2 diabetes mellitus with severe NPDR with macular edema
ICD-10-CM	F10.2	Alcohol dependence
ICD-10-CM	H34.81	Central retinal vein occlusion
ICD-10-CM	H34.83	Tributary (branch) retinal vein occlusion
ICD-10-CM	H34.23	Retinal artery branch occlusion
ICD-10-CM	H34.1	Central retinal artery occlusion
ICD-10-CM	H34.21	Partial retinal artery occlusion
ICD-10-CM	Z59.6	Low income
CPT	67028	Intravitreal injection of a pharmacologic agent (separate procedure)
TNX Curated	9014	Hemoglobin [Mass/volume] in Blood
TNX Curated	9037	Hemoglobin A1c/Hemoglobin. Total in Blood
TNX Curated	9004	Triglyceride [Mass/volume] in Serum, Plasma, or Blood
TNX Curated	9083	Body Mass Index (BMI)
TNX Curated	9000	Cholesterol [Mass/volume] in Serum or Plasma
ICD-10-CM	E11.33	Type 2 diabetes mellitus with moderate NPDR
ICD-10-CM	E11.31	Type 2 diabetes mellitus with unspecified diabetic retinopathy
ICD-10-CM	E11.32	Type 2 diabetes mellitus with mild NPDR
ICD-10-CM	E11.34	Type 2 diabetes mellitus with severe NPDR
TNX Curated	9052	Lactate dehydrogenase [Enzymatic activity/volume] in Serum or Plasma

ICD-10-CM: International Classification of Diseases, 10th Revision, Clinical Modification; CPT: Current Procedural Terminology; TNX: TriNetX curated variable; NPDR: Non-Proliferative Diabetic Retinopathy; BMI: Body Mass Index; LDH: Lactate Dehydrogenase.

**Table 4:** Risk of Proliferative Diabetic Retinopathy and Related Complications by LDH Level.

Comparison	Outcome	Risk (%)–Low LDH	Risk (%)–Comparison Group	Relative Risk (95% CI)
Low vs. Moderate LDH	PDR	2.96%	3.93%	1.33 (1.14–1.54)
Low vs. Moderate LDH	TRD	0.99%	1.35%	1.36 (1.07–1.73)
Low vs. Moderate LDH	VH	3.51%	4.38%	1.25 (1.10–1.42)
Low vs. High LDH	PDR	3.00%	3.66%	1.22 (1.05–1.42)
Low vs. High LDH	TRD	0.96%	1.27%	1.32 (1.04–1.67)
Low vs. High LDH	VH	0.96%	1.27%	1.22 (1.08–1.40)

PDR: Proliferative Diabetic Retinopathy, TRD: Tractional Retinal Detachment, VH: Vitreous Hemorrhage, LDH: Lactate Dehydrogenase, CI: Confidence Interval.

## Data Availability

The data are available upon request.
